# Analysing the characteristics of a measles outbreak in Houaphanh province to guide measles elimination in the Lao People's Democratic Republic

**DOI:** 10.5365/wpsar.2017.8.1.012

**Published:** 2018-07-27

**Authors:** Bounthanom Sengkeopraseuth, Bouaphanh Khamphaphongphane, Phengta Vongphrachanh, Anonh Xeuatvongsa, Sisouveth Norasingh, Chansay Pathammvong, Manilay Phengxay, Phanmanisone Philakong, Siddhartha Sankar Datta

**Affiliations:** a Lao People’s Democratic Republic Field Epidemiology Training Programme.; b National Center for Laboratory and Epidemiology, Lao People’s Democratic Republic.; c National Immunization Programme, Lao People’s Democratic Republic.; d World Health Organization, Lao People’s Democratic Republic.

## Abstract

**Introduction:**

In recent years, the incidence of measles has declined in the Lao People's Democratic Republic. However, an outbreak was reported in August 2014 in Houaphanh province, which was the biggest outbreak in the country since 2008. We describe the characteristics of this outbreak and outline critical interventions for the Lao People's Democratic Republic to achieve measles elimination.

**Methods:**

Fever and rash cases in the Khouan and Samtai districts with an onset date from 1 September to 25 October 2014 were investigated. Active case finding and health facility record reviews were carried out. Appropriate samples from the individuals with suspected measles were tested to confirm the diagnosis.

**Results:**

A total of 265 suspected cases including 12 deaths were reported from eight villages in the Khouan and Samtai districts. Forty-five individuals tested positive for measles IgM. Most of the confirmed patients were male (*n* = 28, 62%), less than 5 years old (*n* = 23, 51%) and from the Hmong ethnic community (*n* = 44, 98%). The majority of the people with suspected measles (*n* = 213, 80%) and all the confirmed ones were unvaccinated. A measles vaccination campaign conducted in the eight affected villages resulted in 76% coverage of the targeted population.

**Discussion:**

Low routine coverage and measles occurrence among unvaccinated individuals indicate underimmunized areas. The geographical and sociodemographic characteristics of this outbreak highlight the need for tailored vaccination strategies to close the immunity gap. A sensitive surveillance system that is able to detect, notify, investigate and guide response measures, including a second measles dose in the routine immunization schedule, will be essential for the Lao People's Democratic Republic to attain its measles elimination status.

## Introduction

Measles is a highly contagious disease caused by measles virus (genus *Morbillivirus*, family *Paramyxoviridae)* and remains one of the most contagious diseases of humans. (*1*) Measles is characterized by rash, fever and cough, coryza or conjunctivitis and is usually transmitted from four days before to four days after the onset of rash. (*1*) The incubation period is normally 10–14 days and complications include otitis media, laryngotracheobronchitis, pneumonia, diarrhoea, encephalitis and secondary bacterial infections. (*1*) Since 1974, the use of safe and cost-effective measles vaccines has resulted in a marked decrease in measles cases and deaths. Globally, measles-related deaths have declined from about 548 300 in 2000 to an estimated 114 900 in 2014. (*2*, *3*) The reduction in measles deaths is a testament to the importance of measles vaccination to global health. However, globally, measles still remains one of the leading causes of death among children under 5 years of age, especially in countries with limited health infrastructure. (*4*-*6*)

Both the Global Vaccine Action Plan endorsed by the World Health Assembly in 2012 and the Global Measles and Rubella Strategic Plan 2012–2020 include elimination of measles, rubella and congenital rubella syndrome as one of the main objectives. (*7*, *8*) All World Health Organization (WHO) regions have established goals to eliminate measles by 2020. (*3*) In 2005, the WHO Regional Committee for the Western Pacific resolved that the Region should aim to eliminate measles by 2012. Elimination is defined as the absence of endemic measles virus transmission in a defined geographical area more than 12 months in the presence of a well performing surveillance system. (*9*) In 2012, the Regional Committee reaffirmed its commitment to eliminate measles. (*10*) The number of measles cases in the Region has decreased from 54 291 in 2009 to 8524 in 2012, and measles incidence decreased by 83% during the same period. (*11*)

The national policy in the Lao People's Democratic Republic is to provide one dose of measles vaccine to all children at 9 months of age. Since 2011, a combination measles-rubella vaccine has been used to provide additional rubella protection for the children. (*12*) Since 2000, the Lao People's Democratic Republic has been providing a second opportunity for measles vaccination to children through periodic supplementary immunization activities (SIAs). (*13*)

Despite reduction in measles incidence since the start of the measles immunization in 1979, (*14*) several sporadic and widespread measles outbreaks have been reported in the Lao People's Democratic Republic. A measles outbreak was reported in August 2014 in Houaphanh province. The outbreak in Houaphanh province outlined the challenges that lay ahead for the country towards achieving measles elimination. We describe the characteristics of the outbreak with the aim of identifying critical interventions for the Lao People's Democratic Republic to attain the measles elimination status.

## Methods

The WHO-accredited National Center for Laboratory and Epidemiology (NCLE) has been receiving weekly reports of measles cases from all provinces in the Lao People's Democratic Republic since 1994. (*14*) On 19 September 2014, Khouan District Health Office received reports of fever, cases of rash and red eyes from Khorhai village, Khouan district, Houaphanh province. The patients were treated at the Khouan and Samtai district hospitals. On 24 September 2014, the District Health Office reported the event to the Houaphanh Provincial Health Office, which subsequently reported the cases to NCLE. Rapid response teams conducted investigations of the suspected cases in Khorhai and neighbouring villages within two days of the notification; the suspected patients attending hospitals were investigated by the physicians. Active case finding through house-to-house visits was conducted in all the affected villages to identify and investigate the suspected cases and ensure initial treatment and referral of patients with measles, as needed, to prevent any deaths. The medical records were reviewed at the provincial and district hospitals including the health centres serving the affected geographical areas for a period of nine months preceding the outbreak to identify any missed cases. The standardized case investigation form and line list routinely used by NCLE to record any infectious disease outbreak was adapted to record the information of these fever and rash cases. The data captured in the line list included the characteristics of each of the suspected cases (which conformed to the measles surveillance case definition) such as age, sex, ethnicity, geographical location, date, type and pattern of rash onset, symptoms, travel history, history of contact with individuals with confirmed measles, outcome of the infection and immunization status.

The identified fever and rash cases were classified according to the WHO standard case definition for measles and rubella. (*15*) A suspected measles case is defined as any person with fever and maculopapular rash (non-vesicular) and cough, coryza or conjunctivitis or any person in whom a clinician suspects measles infection. A laboratory-confirmed measles case is one that meets the above case definition and the presence of measles-specific IgM antibodies is confirmed in a WHO-accredited laboratory.

The vaccination status was reported either by the patient, caregiver or parents present at the time of the investigation. The childhood vaccination card when available was checked to ascertain the reported vaccination status of the cases. Serum samples could not be collected from the suspected cases detected retrospectively during the active case finding or from those who were not in the appropriate time frame for sample collection. To ensure the reliability of the laboratory tests performed to confirm the diagnosis of measles, the optimal time frame of between 7–28 days from the date of rash onset for specimen collection was ensured by the investigation team during the collection of the serum samples. (*16*) WHO recommends that a single serum sample be obtained from suspected measles cases at the first contact with the health-care system within 28 days after rash onset for adequate measles surveillance; IgM ELISA detection is most sensitive 4–28 days after the rash onset. (*17*) The serum samples were sent to NCLE for measles and rubella IgM testing using ELISA (Enzygnost® kits, Siemens, Erlangen, Germany). Viral genotyping was carried out at the Public Health Laboratory Centre, Department of Health, Hong Kong Special Administrative Region SAR (China).

Affected districts reported all suspected cases to NCLE daily for a 14-day period from 26 September to 10 October 2014.

## Results

### Geographical distribution of cases

Over a period of eight weeks, 265 suspected cases including 12 deaths (case fatality rate: 4.5%) were reported from four adjoining villages in the Khouan and Samtai districts in Houaphanh province. Of the 265 suspected cases, 45 were laboratory-confirmed. All deaths resulted from pneumonia, and all case-patients had fever and rash. Most of the confirmed cases (*n* = 34, 76%) and the deaths (*n* = 9, 75%) were reported from Khouan district. Most of the confirmed cases were reported from Khorhai (*n* = 15, 33%) and Houiybeuy (*n* = 11, 24%) villages in the Khouan district, while the rest of the cases were located in six different villages in both districts.

The cases that were suspected but not confirmed (*n* = 214) were reported mainly from Khorhai village, Khouan district (*n* = 94, 44%), while the rest of the 51 cases were located in 16 different villages of both districts. The majority of the suspected case-patients in Khouan district belonged to the Hmong ethnic community (*n* = 211, 80%). All suspected case-patients had fever and rash, 54% (*n* = 143) had cough and 41% (*n* = 109) had runny noses at the time of investigation.

### Characteristics of confirmed cases

Most of the 45 people with confirmed measles were male (*n* = 28, 62%), ranging from 6 months to 18 years of age. The median age of patients with confirmed measles was 5 years. The highest proportion of the confirmed patients (*n* = 23, 51%) were less than 5 years of age followed by those 5–9 years old (*n* = 17, 38%). Similar age characteristics were also noticed among the suspected case-patients. All infants less than 1 year old with confirmed measles were less than 9 months old.

The majority (*n* = 44, 98%) of people with confirmed measles were from the Hmong ethnic community, and only one patient (*n* = 1, 2%) was reported from the Laolum ethnic community. All four confirmed patients from Hinteng village in the Khouan district and Phanhsavanh village in Samtai district had travelled to Khorhai village of the Khouan district, which reported the highest number of confirmed cases.

**Fig. 1:**
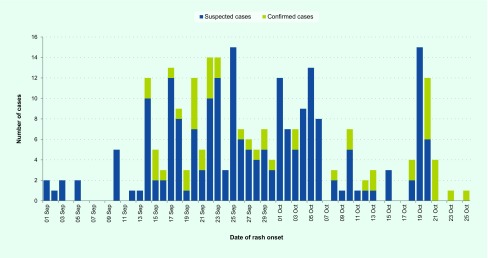
Epidemiological curve of measles cases during the outbreak investigation, Houaphanh province, Lao People's Democratic Republic, 1 September–25 October 2014

The epidemiological curve of the outbreak shows a classical pattern of a propagated source of person-to-person transmission (**Fig. 1**).

### Clinical characteristics of confirmed cases

All patients with confirmed measles reported fever and rash. The rashes were identified as maculopapular in 87% (*n* = 39) of the confirmed patients. The other symptoms reported by the confirmed patients were cough (*n* = 36, 80%), conjunctivitis (*n* = 36, 80%), runny nose (*n* = 29, 64%) and diarrhoea (*n* = 10, 22%). Of the 30 confirmed case-patients who were hospitalized, 29 were admitted to the district hospitals. Most (90%) of the hospitalized patients (both suspected and confirmed patients) were less than 10 years of age. Six (20%) of these hospitalized patients developed pneumonia. There were no reported cases of encephalitis.

### Vaccination status

None of the confirmed patients and only one (0.5%) of the suspected case-patients had received any dose of measles-containing vaccine by the time of the investigation. The characteristics of the suspected and confirmed patients are shown in **Table 1**.

**Table 1:** Characteristics of measles patients, Houaphanh province, Lao People's Democratic Republic, 2014

Characteristics	Suspected but not confirmed (*n* = 214)		Laboratory confirmed (*n* = 45)		Tested “equivocal” (*n* = 6)		Total (*n* = 265)	
	n	%	n	%	n	%	n	%
**Sex**								
**Male**	**105**	**49**	**28**	**62**	**2**	**33**	**135**	**51**
**Female**	**109**	**51**	**17**	**38**	**4**	**67**	**130**	**49**
**Age group (years)**								
** < 1 year**	**11**	**5**	**3**	**7**	**0**	**0**	**14**	**5**
**1–4 years**	**97**	**45**	**20**	**44**	**1**	**17**	**118**	**45**
**5–9 years**	**80**	**38**	**19**	**43**	**4**	**66**	**103**	**39**
**10–14 years**	**22**	**10**	**2**	**4**	**1**	**17**	**25**	**9**
** ≥ 15 years**	**4**	**2**	**1**	**2**	**0**	**0**	**5**	**2**
**Vaccination status**								
**None**	**213**	**99.5**	**45**	**100**	**6**	**100**	**264**	**99.6**
**Yes**	**1**	**0.5**	**0**	**0**	**0**	**0**	**1**	**0.4**
**Case type**								
**Suspected but not confirmed**	**-**						**214**	**80.70**
**Laboratory confirmed**							**45**	**16.98**
**Laboratory tested but results “equivocal”**							**6**	**0.02**

### Laboratory diagnosis

Serum samples were collected from 51 (19%) of the 265 patients with suspected measles; in addition, nasopharyngeal swabs were collected from only nine suspected patients (3%). Forty-five patients (88%) tested positive by ELISA for measles-specific IgM antibodies; the test results in the remaining six patients were equivocal. All but one sample tested negative for rubella-specific IgM antibodies. The genotype of the detected measles virus identified in this outbreak was H1.

### Vaccination response in the area

The immunization programme units of the Samtai and Khouan districts carried out outbreak response immunizations targeting individuals between 9 months and 20 years following detection of the confirmed cases. Approximately 19 600 children, irrespective of their previous vaccination status, were vaccinated with one dose of measles-rubella vaccine, achieving an overall 76% coverage in the targeted villages with Samtai district achieving higher coverage (84%) than the Khouan district (63%).

## Discussion

The suboptimal vaccination status of the confirmed measles cases in the affected areas indicates pockets of underimmunized populations in the Lao People's Democratic Republic. Since the Lao People's Democratic Republic has adopted the goal of measles elimination as part of a 2005 WHO Regional Committee resolution, (*9*) the population immunity needs to be sustained above 95% in all districts of the country to prevent measles epidemics. (*1*)

Measles epidemics have been reported in communities with low vaccination coverage. (*18*) The coverage of the first dose of measles-rubella vaccine in Khorhai in 2013 was around 50% [reports from National Immunization Programme, unpublished data]. The vaccination coverage for the previous years in Khorhai and in other affected villages could not be assessed as the monthly vaccination records could not be retrieved from the health centres. The administrative coverage of the first dose of measles-containing vaccine (MCV1) of Samtai district was 45%, 49% and 24% for 2011, 2012 and 2013, respectively, while the MCV1 coverage of Khouan district was 52% for 2013 [reports from National Immunization Programme, unpublished data]. Khouan is a newly created administrative district and hence the vaccination coverages for the years 2011 and 2012 are not available. The reported administrative MCV1 coverage of the Houaphanh province was around 59% in 2011, 2012 and 2013 [reports from National Immunization Programme, unpublished data], while the reported coverage for the national level was 69%, 72% and 82% for the corresponding years. (*19*)

Because MCV1 in the Lao People's Democratic Republic is provided at 9 months of age, the country should consider adding a routine second dose of measles-containing vaccine at age 15–18 months to reduce the rate of accumulation of susceptible children and risk of a future outbreak. (*1*) Countries aiming for measles elimination should achieve and maintain greater than 95% coverage with two doses in every district of the country. (*1*)

Because of the delay in reporting by the health centre, almost three weeks after the occurrence of the first case, and the fact that only 19% of the suspected cases had a serum sample, the results of our outbreak investigation may not truly estimate the actual burden of measles in this area.

Houaphanh province is located in the eastern part of the Lao People's Democratic Republic. It has eight administrative districts and is one of the poorest provinces with a total population of around 310 000. (*20*) The terrain is rugged with dense, mountainous forest forming much of the land mass. The affected villages, including Khorhai village, are situated around 15 km and 30 km from the nearest health centre and the district headquarters, respectively. All eight affected villages are primarily mountainous and have poor road conditions; more than half of the roads are inaccessible during the rainy season, making it difficult for the local health centres to deliver routine vaccination services. These geographic difficulties also make it difficult for these villagers to access the health-care services in the nearest health centres.

Countries with weaker health infrastructure or areas within the countries with moderate or weak functioning health system have used SIAs to deliver measles vaccine to children who were missed by routine vaccination or who are outside the health system. SIAs have been used in the Lao People's Democratic Republic since 2011. (*13*) The effectiveness of SIAs in reaching the vulnerable population in the Lao People's Democratic Republic should be evaluated. Studies have shown that in situations with low routine immunization coverage, measles vaccination through supplemental immunization using outreach activities helps reduces the accumulation of susceptible people and is cost effective. (*21*, *22*) The risk of measles outbreaks is determined by the rate of accumulation of the susceptible population; (*1*) thus, the National Immunization Programme of the Lao People's Democratic Republic should routinely analyse the available coverage data and immunity gap to monitor the accumulation of susceptible people and plan follow-up SIAs.

Ninety-eight per cent of the patients with measles were reported from the Hmong community, illustrating that an immunity gap exists in this group. The high case fatality seen in this outbreak is comparable to the fatality rate seen in the past outbreaks. (*14*) The national measles vaccination coverage in the Lao People's Democratic Republic Social Indicator Survey 2011–12 was 55.3% with a wide disparity of vaccination coverage between the ethnic communities, ranging from 35.3% in the Hmong-Mien community to 72.7% in the Lao People's Democratic Republic-Tai community. (*23*) Data about vaccination coverage in ethnic groups are not routinely collected by the immunization programme and are available only from periodic national coverage surveys.

The outbreak has primarily affected unvaccinated children less than 5 years old who should have received their vaccination doses during routine immunization or during the periodic SIAs conducted in the Lao People's Democratic Republic. A wide-age-range (9 months to 19 years) measles-rubella SIA was conducted in the Lao People's Democratic Republic in 2011, (*23*) but this outbreak indicates that these cases had missed both the routine and SIA doses. A similar pattern of age-group affected, rate of pneumonia and low vaccination status was observed during a measles outbreak in a district of Balochistan province of Pakistan where the affected population had difficulties in accessing health facilities and had poor routine vaccination coverage. (*24*)

The H1 genotype identified in this outbreak was also detected in the Lao People's Democratic Republic in 2011–2012 and has been the predominant genotype detected in China between 2009 and 2012. (*11*, *25*)

There were several limitations to the investigation of this outbreak. The health-seeking behaviour of the community and their knowledge about measles were not assessed to understand the reasons of low routine coverage in the affected villages. The reported vaccination status of the cases were not verified with the immunization registers at the health centres, and thus recall biases would be inevitable. The existing system of passive notification of measles cases could still be useful for decision-making if the information were promptly shared with the district and provincial levels by the reporting health facilities. (*26*) The delay in timely reporting resulted in the health system taking 41 days to conduct any SIAs after the detection of the suspected cases in the community. While WHO recommends the use of serum-based IgM ELISA assays to confirm clinically suspected measles/rubella, (*1*, *15*, *23*) there is an inherent limitation in using IgM ELISA for confirmation of measles when the serum samples are collected within four days of rash onset. However, this may not have been relevant in an outbreak setting as individual diagnosis is not critical. (*17*) In this outbreak, 22% of the serum samples were collected within four days of rash onset; however, all samples tested either positive or equivocal for measles IgM by ELISA. The sample collection for measles diagnosis should be further improved by collecting urine or nasopharyngeal samples to confirm the outbreaks and document measles elimination by virus genotyping. (*17*, *27*) Lastly, the line list prepared in this outbreak did not indicate the method of case detection; hence, the differentiation of the cases identified during active case finding or in routine surveillance was not possible.

This outbreak in Houaphanh province was the biggest measles outbreak in the Lao People's Democratic Republic since 2008. The outbreak highlights the vulnerability of the ethnic and other geographically dispersed communities in the country to any vaccine-preventable diseases. To achieve measles elimination, the National Immunization Programme should consider investing in ways to identify and target high-risk populations and use community-specific strategies to close immunity gaps. This includes regular outreach activities and the introduction of a second dose of measles vaccine in the national immunization schedule. To achieve elimination, it is crucial that a sensitive surveillance system that can detect, notify and ensure timely investigation of suspected cases, classify them as confirmed or discarded and guide appropriate response measures to prevent further transmission in the Lao People's Democratic Republic.
